# Kinins and Kinin Receptors in Cardiovascular and Renal Diseases

**DOI:** 10.3390/ph14030240

**Published:** 2021-03-08

**Authors:** Jean-Pierre Girolami, Nadine Bouby, Christine Richer-Giudicelli, Francois Alhenc-Gelas

**Affiliations:** 1 INSERM U1138, Centre de Recherche des Cordeliers, 75006 Paris, France; jeanpierregirolami@gmail.com (J.-P.G.); nadine.bouby@inserm.fr (N.B.); christine.giudicelli@free.fr (C.R.-G.); 2 Rangueil Medical School, Université Paul Sabatier, 31062 Toulouse, France; 3 Descartes Medical School, Paris-Université, 75006 Paris, France; 4 School of Sciences and Engineering, Sorbonne-Université, 75006 Paris, France

**Keywords:** kallikrein, kinins, angiotensin-converting enzyme/kininaseII, kinin receptors, arteries, heart, kidney, ischemic heart disease, diabetes

## Abstract

This review addresses the physiological role of the kallikrein–kinin system in arteries, heart and kidney and the consequences of kallikrein and kinin actions in diseases affecting these organs, especially ischemic and diabetic diseases. Emphasis is put on pharmacological and genetic studies targeting kallikrein; ACE/kininase II; and the two kinin receptors, B1 (B1R) and B2 (B2R), distinguished through the work of Domenico Regoli and his collaborators. Potential therapeutic interest and limitations of the pharmacological manipulation of B1R or B2R activity in cardiovascular and renal diseases are discussed. This discussion addresses either the activation or inhibition of these receptors, based on recent clinical and experimental studies.

## 1. Introduction

The plurality of structurally related but molecularly distinct membrane receptors triggering cellular signaling and physiological action is a feature of several vasomotor peptide systems, such as renin–angiotensin, kallikrein–kinin and vasopressin. The receptor responsible for the main physiological action of the peptide was discovered first, and then other subtypes were identified, through refined pharmacological or genomic studies. The physiological role of the “secondary” subtypes depends on their distribution and relative abundance in organs compared to the main receptor and also on coupling to specific cellular signaling pathways. In the case of the renin–angiotensin system, the AT2 receptor for angiotensin II mediates vascular effects roughly opposite to those triggered by the AT1 receptor. However, angiotensin II is mainly a vasoconstrictor in physiological condition, through AT1 receptor activation, and the physiological role of the AT2 receptor remains incompletely understood [1,2].

For the kallikrein–kinin system, thanks to the work of Domenico Regoli, a second receptor for kinins was discovered in addition to the main one mediating the endothelial, epithelial and neuronal action of bradykinin or its human counterpart, lysyl–bradykinin. This second kinin receptor, paradoxically called B1 (B1R) in nomenclatures, interestingly has higher affinity for a bradykinin fragment, desArg9-BK, than for native BK. Activation of the B1 receptor thus depends on the availability of both kinins and carboxypeptidases hydrolyzing BK and releasing desArg9-BK. Another interesting feature of the B1 receptor is its absence or low abundance in resting condition but its inducibility in pathological situations by several physicochemical and biological factors that include hypoxia, ischemia and hyperglycemia. It was through these peculiar properties that the B1 receptor was discovered by Regoli and collaborators [3,4]. The main receptor mediating the vasodilator action of bradykinin, called B2 (B2R), on the other hand, is constitutively synthesized and present in abundance in the vascular endothelium and other tissues. This receptor binds bradykinin and lysyl–bradykinin with favorable kinetic properties and has high signaling efficacy [4,5]. Both B1R and B2R are structurally related, coded by two, partly homologous neighboring genes [6]. Coupling to cellular signaling pathways does not seem to differ significantly between the two receptors, at least in cellular models. However, this remains to be further studied in pathological conditions where B1R or B2R activation may have different pathophysiological consequences, as discussed below.

Bradykinin is a potent endothelium activator and vasodilator coupled to nitric oxide synthase (NOS) and nitric oxide, Phospholipase A2 (PLA2) and its products, such as prostacyclin and hyperpolarizing eicosanoid factors [7]. The activation of NOS and PLA2 by bradykinin in endothelial cells of arteries, including coronary arteries, results in the production of several chemical and biochemical compounds, nitric oxide, prostacyclin and other eicosanoids, relaxing by paracrine action vascular smooth muscle cells [8,9,10]. These mediators also inhibit, on the endothelial surface and in blood, platelet aggregation. In addition, bradykinin stimulates the release of plasminogen activator by the endothelium, thereby promoting fibrinolysis [11]. The role of kinins in thrombosis remains, however, poorly documented. Genetic and pharmacological studies focused on components of the kallikrein–kinin system (kallikrein, kinin receptors and angiotensin-converting enzyme/kininase II, ACE) have shown that kinins are formed endogenously through the action of tissue-kallikrein, degraded by ACE, and participate through B2R activation in arterial physiology, controlling blood flow delivery to organs. In several pathological situations, such as ischemia or chronic hyperglycemia, kinin actions afford end-organ protection, especially in the heart and kidney. On the other hand, excess bradykinin in the circulation, locally or systematically, may become a life-threatening situation, illustrated by angioedema or endotoxin-shock. This is usually achieved through inappropriate activation of a latent kinin-forming protease only present in plasma, called plasma (pre)kallikrein.

In line with the “Janus faces” of kinins in disease and their protective or pathogenic effects, depending mainly on local abundance, pharmacological interventions aimed at blocking or, inversely, activating kinin receptors have been developed. This review addresses the physiological or pharmacological agonism and antagonism of kinin receptors, B1R or B2R, in clinical and experimental diseases.

## 2. Physiological Role of Endogenously Produced Kinins

Kinins are released from plasma kininogens in the circulation and interstitium of organs synthesizing kallikrein. Kallikrein-synthesizing organs include large or small arteries, the heart, the kidney and other exocrine glands, the intestine and the central nervous system [12,13]. The physiological role of endogenously produced kinins has been well documented in the cardiovascular system and the kidney, in both mice and humans [14]. This was achieved through the study of mice or humans genetically deficient in kallikrein activity or B2 receptor and in animals treated with a B2R antagonist [15,16,17,18,19].

Kinins are not involved in the regulation of systemic blood pressure but participate in other aspects of arterial physiology, especially flow-mediated vasodilatation, a critical feature of arterial function, which is endothelium mediated, ensuring the proper delivery of blood to organs [15,20,21,22]. In the kidney, kallikrein and/or kinins are also involved in electrolyte transfer in the distal nephron, where kallikrein is synthesized in epithelial cells in the connecting tubule [23,24,25,26]. The vascular physiological actions of kinins are B2R mediated [4,27]. The B1R does not seem to play a significant role in cardiovascular and renal physiology in healthy animals. Some of the renal actions of kallikrein may not be kinin-mediated [19].

In several experimental pathological situations, deficiency in kallikrein and kinins, or in B2 receptor aggravates end-organ damage. This has been well established in the settings of cardiac, renal or peripheral ischemia, and in diabetes. The synthesis of kallikrein and kinin receptors in heart or kidney is stimulated by ischemia or chronic hyperglycemia [28,29]. Kinins then exert several B2R-mediated actions affording tissue protection, such as vasodilation of collateral blood vessels, reduction in oxidative stress and stimulation of post-ischemic angiogenesis [30,31,32,33]. Loss of function studies suggest that, through these actions, kinins reduce infarct size in acute cardiac ischemia and, in post-ischemic heart failure, prevent excess ventricular remodeling, severe hemodynamic failure and death [28,34]. Kinins also have an organ-protective role in kidney ischemia reperfusion [35]. In peripheral, hindlimb ischemia, kallikrein and kinins promote vasculogenesis and accelerate the recovery of distal blood perfusion [32,36]. In the diabetic kidney, kallikrein and kinins reduce hyperglycemic kidney damage and slow nephropathy progression [14,25].

The effects of kinins in ischemia or diabetes are mainly B2R mediated [18,28], but the BIR, which is induced in these pathological situations, may also be involved. B1R was suggested to exert organ-protective actions in the ischemic heart and kidney but not the brain or intestine [35,37,38,39,40]. Interestingly, a deficiency in B2R in insulinoprive diabetic Akita mice not only aggravates renal damage but also induces a generalized pro-senescent phenotype [41].

The issues of kinin actions and kinin receptor roles in ischemia and diabetes are further considered below when discussing gain-of-function studies.

## 3. Pharmacological Activation of Kinin Receptors

### 3.1. Kinins as Therapeutic Agents in ACE/Kininase II Inhibitor or Angiotensin II AT1 Receptor Blocker Treatment

ACE, or kininase II, is the main enzyme inactivating kinins in the circulation [42]. ACE also activates angiotensin I into angiotensin II. ACE inhibitors were originally designed for lowering blood pressure in hypertensive subjects by interrupting the renin-angiotensin system [43]. These inhibitors were subsequently shown to be also very efficient clinically in several pathological situations where excess vasoconstriction, locally and/or systematically, is detrimental, such as heart failure or diabetic nephropathy. They were eventually further shown to prevent major cardiovascular events in high-risk subjects [44].

Both animal and human studies have documented increases in kinin levels in blood and tissues during ACE inhibitor treatment [45,46]. The role of kinins in the therapeutic effect of ACE inhibitors has been addressed in numerous studies in genetic animal models of kallikrein or kinin receptor deficiency and in animals treated concomitantly with a kinin B2R antagonist. Kinins are not involved in the blood pressure lowering effects of the drugs in hypertension. This is consistent with these peptides being autacoids produced and destroyed locally in selective organs rather than systemically acting vascular hormones. However, studies have consistently shown that the inhibition of kinin formation or action dramatically reduces or even abolishes the beneficial effect of ACE inhibitors in experimental cardiac ischemia, post-ischemic heart disease and also in peripheral ischemia (for review [47,48]). Similar observations have been made in experimental diabetic nephropathy, although the role of kinins in the effect of ACE inhibitors may be greatest at the early stage of the disease [49,50,51,52,53]. These observations should be extrapolated with caution to human diseases and their treatment but nevertheless support that kinins are involved in the multiple beneficial effects of ACE inhibitors in the human heart and kidney.

The renin–angiotensin was subsequently targeted by developing orally active angiotensin II AT1 receptor blockers (commonly designed as sartans, [54]). These drugs are also efficient in hypertension, heart failure and diabetic nephropathy. The issue of their relative efficiency compared to ACE inhibitors has been addressed in clinical trials but not definitely resolved [55]. Probably, in clinical practice, both classes of drugs can be considered to be equally efficient in most indications. However, interestingly, sartans do work, at least in part, through kinin release and B2R activation, like ACE inhibitors but by a different mechanism. The arterial, cardiac and renal effects of AT1 receptor blockade can indeed be suppressed by kallikrein or kinin receptor deficiency or pharmacological kinin B2R blockade. This has been well documented in healthy animals and in experimental cardiac ischemia or post-ischemic heart failure [56,57,58]. These observations linking sartans to kinins, originally made in the kidney, have documented a physiological coupling between the AT2 receptor for angiotensin II, which is activated during AT1 receptor blockade, and the kallikrein–kinin system [59]. This coupling involves kallikrein, kinin release and also, possibly, the direct molecular interaction between AT2 and B2 receptors [56,57,60].

Overall, the studies with ACE inhibitors or sartans document the therapeutic action of endogenous kinins in cardiovascular and renal diseases, when the bioavailability of the peptides is enhanced by the pharmacological stimulation of their production, or diminished inactivation. These beneficial effects of kinins are B2R mediated. However, during ACE inhibitor treatment, the interesting observation has been made that the B1R is induced, at least in the kidney [61]. Molecular mechanisms underlying this induction remain obscure. However, the consequence is that B1R activation occurs during ACE inhibitor treatment and may participate, together with B2R activation, in the therapeutic actions of the drug.

While kinins are involved in the therapeutic action of ACE inhibitors and AT1 receptor blockers, they may also be responsible for some unwanted effects of these drugs, cough and angioedema [62]. Cough is relatively frequent under ACE inhibitors. It has been attributed to effects of kinins in the tracheo-bronchial tractus. However, ACE inhibitor-induced cough is dry and may instead be caused by substance P, another known ACE substrate. Dry cough is best reproduced experimentally in animals by administering substance P. However, kinins have been shown to stimulate the release of substance P in tracheal nerves and may thus be, indirectly, involved in cough [63]. On the other hand, kinins are most likely involved in angioedema occurring under ACE inhibitors, although the curative effect of a B2R antagonist has not been consistently observed in all studies in the iatrogenic contrary to the idiopathic form of the disease (see below).

Inhibitors of the metalloendopeptidase neprilysin have also been developed for the treatment of heart failure. The rationale for this development was based on the role of neprilysin in inactivating natriuretic peptides. However, neprilysin is also a secondary kininase in the circulation, and its inhibition increases kinin levels [64]. Neprilysin inhibitors have been found to be well tolerated but poorly effective in treating heart failure. A mixed ACE-neprilysin inhibitor, omapatrilat, has been designed and tested in clinical trials but eventually not approved for clinical use because of its poor tolerance with a high incidence of angioedema [65]. Recently, a drug mixing an angiotensin II AT1 receptor antagonist (valsartan) and a neprylisin inhibitor (sacubitril), LCZ696 or Entresto, was found to be very effective in the treatment of congestive heart failure with reduced ejection fraction [66]. The drug has received regulatory approval in this indication. Tolerance was acceptable with an incidence of angioedema not superior to other drugs used in the indication, including ACE inhibitors [64]. LCZ696 should increase the bioavailability of kinins through both its angiotensin II AT1 receptor blocking and neprylisin inhibiting actions. However, data on LCZ696 and kinins are still missing, and the role of kinins in the therapeutic effect of LCZ696 remains to be investigated.

The concept of kinin receptor activation as a therapy for cardiovascular and renal diseases was further documented by studying pharmacological agonists of kinin receptors.

### 3.2. Direct Pharmacological Agonism of Kinin Receptors

For pharmacological agonism of the kallikrein–kinin system, either kallikrein or kinin receptors can be targeted. Kallikrein cannot be easily used as a therapeutic agent for pharmacokinetic reasons, although some animal studies have been conducted in diabetes or cerebrovascular diseases with the native or recombinant enzyme [67]. Kallikrein has received regulatory approval in China for treating stroke, but its efficacy has not been documented by appropriately conducted clinical trials. Aliskiren, a compound originally designed as a renin inhibitor, has been reported to stimulate cardiac kallikrein synthesis in the rat and reduce ischemia-reperfusion injury through kinin release and B2R activation [68]. However, the drug has additional pharmacological actions.

Kinin receptors have long been targeted with synthetic bradykinin analogs designed for inhibiting kinin binding and receptor activation. Analogs with agonist rather than antagonist properties have been identified in the course of this research. Some compounds were designed by Gobeil, Regoli and collaborators [69,70]. These molecules are pseudo-peptides that should be administered parenterally. They are selective B1R or B2R, resistant to peptidases and pharmacodynamically potent.

The B2R agonist dose-dependently decreases blood pressure in healthy animals after acute administration but does not retain its hypotensive effect in chronic administration, most likely because of hemodynamic counter-regulations. The B1R agonist has no effect on blood pressure [71].

These agonists are useful tools for further documenting the effects of kinins on health and disease, probing the role of B1R and B2R and documenting the cellular signaling pathways involved. The agonists have been initially used in animals for enhancing drug delivery to the brain by opening the blood–brain barrier [72,73]. The compounds have also been studied in experimental ischemic and diabetic diseases.

**Figure 1:**
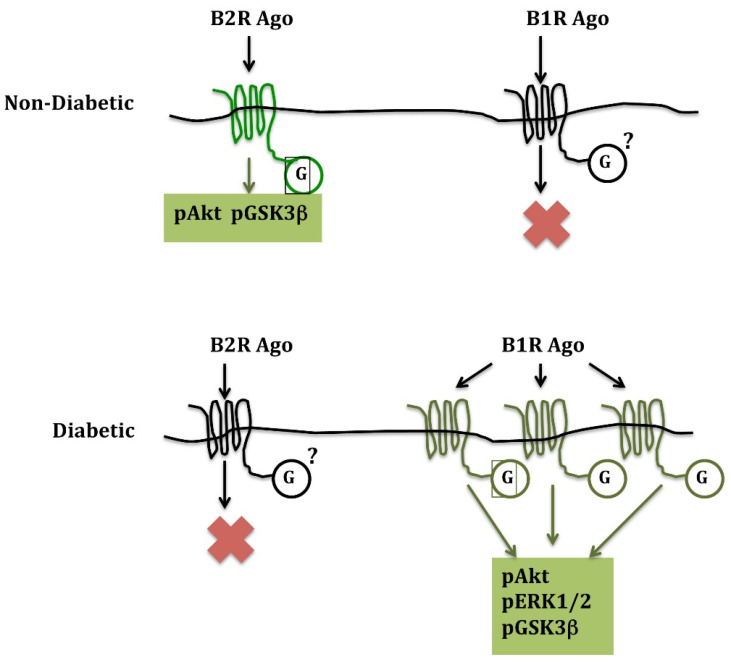
Alternate use of B2R and B1R in cardioprotection in non-diabetic and diabetic mice, respectively, during cardiac ischemia-reperfusion. B1R ago: pharmacological B1R agonist; B2R ago: pharmacological B2R agonist. G: G proteins; pAkT: phosphoinositide 3 kinase/Akt; pERK1/2: extracellular signal-regulated kinase1/2; pGSK3b: glycogen synthase-kinase3; p is for phosphorylated forms of the enzymes. Note that B1R but not B2R synthesis increases more than three times in the diabetic heart. Infarct size-reducing effect is associated with activation of the so-called “Reperfusion Ischemia Salvage Kinase (RISK)” pathway and inhibition of GSK-3β. Based on data presented in [71].

In acute cardiac ischemia and ischemia-reperfusion, a B2R agonist given at reperfusion dramatically (45%) reduces infarct size in non-diabetic mice. A B1R agonist has no effect despite the synthesis of B1R in the ischemic heart. However, in diabetic animals, the opposite is observed: the B2R agonist loses its cardio-protective effect, while the B1R agonist reduces infarct size by 43% [71]. These observations are consistent with B2R signaling becoming inactive in the diabetic heart and B1R, which is induced in diabetes, taking over cardio-protective signalization. This signalization involves activation of the phosphoinositide 3 kinase/Akt pathway, leading to the inhibition of glycogen synthase kinase-3β, for both B1R and B2R [71] (Figure 1).

These, as well as other studies [74], further document, through a gain of function approach, the cardio-protective effect of kinins in ischemia. They also unravel a peculiar effect of diabetes on cardiac signaling, switching over cardio-protective signaling from B2R to B1R. Interestingly, in the diabetic and ischemic mouse heart, the B1R agonist was the only treatment reducing infarct size, as an ACE inhibitor or ischemic post-conditioning was without effect [71]. The mechanism of diabetes effect on the kinin receptor signaling pathways remains unknown. It may be related to the differential abundance of G proteins and kinases in the non-diabetic and diabetic heart, but this is speculative.

The substitution of B1R for B2R signaling with similar physiological consequences has also been observed after *B2R* gene inactivation in mice. In the absence of B2R, B1R is induced and takes over kinin signalization, in both arteries and heart [28,75]. The mechanism of B1R induction after *B2R* gene inactivation remains unknown. Overall, these observations document potential redundancy in kinin signalization. Redundancy in signalization may explain in part the difficulty encountered in delineating the respective role of each receptor in some pathological situations. Indeed, the issue has generally been addressed experimentally by inactivating one receptor, B1R or B2R, genetically or pharmacologically, and assuming that the phenotype observed is solely caused by loss of function of the targeted receptor. However, compensation by the other receptor, if occurring, may introduce bias in the interpretation of data.

Another example of the influence of diabetes on kinin signaling and action is brain ischemia-reperfusion. Both B1R and B2R are synthesized in the ischemic brain. In brain ischemia-reperfusion, a B2R agonist has deleterious effects, increasing early mortality, probably through peripheral vascular effects potentiating hemodynamic instability. B1R activation has no effect on mortality or infarct size. However, in diabetic animals, while the B2R agonist increases mortality like in non-diabetic animals, the B1R agonist reduces infarct size and improves neurological deficits [76]. These data should be extrapolated with caution to human cerebrovascular disease but suggest that kinins can afford brain protection against ischemia through B1R activation when produced locally but if released in the circulation aggravate the condition, through peripheral B2R activation.

In peripheral ischemia secondary to femoral artery ligation, a B1R or B2R agonist administered by osmotic minipumps for two weeks after ligation stimulates post-ischemic angiogenesis and accelerates distal perfusion recovery [77]. These studies were performed in diabetic mice because non-diabetic animals quickly recover distal blood perfusion after femoral artery ligation and are not a good model for studying pro-angiogenic treatments. On the contrary, diabetic animals have a defect in post-ischemic angiogenesis. Interestingly, either a B1R or B2R agonist corrects this defect, similarly, and restores the defective angiogenesis in diabetic animals.

Delayed skin wound healing is a complication of peripheral ischemia in human diabetes and can lead to the development of foot ulcers. The effect of B1R or B2R receptor activation on wound healing was studied in (non-ischemic) diabetic or non-diabetic mice. B1R and B2R mRNAs increase in the diabetic wounded skin. The B2R agonist, administered systematically, delays wound healing, probably through both pro-inflammatory and epithelial antiproliferative actions [78]. On the other hand, an antagonist of B2R, icatibant, improves wound healing in diabetic animals and thus is a potential treatment for diabetic foot ulcers. The B1R agonist has no effect, beneficial or detrimental, on the wounded skin, whether in diabetic or non-diabetic animals.

Kinin receptor agonists must still be studied in kidney diseases, ischemic or diabetic. This might be rendered difficult by the relative resistance of the mouse kidney to established nephroprotective treatments, including ACE inhibitors and AT1 receptor blockers.

Overall, while a B2R agonist displayed beneficial effects on the ischemic heart or limb but detrimental effects on the ischemic brain or wounded skin, a B1R agonist consistently displayed beneficial effects in the ischemic heart, brain or hindlimb, in diabetic animals.

## 4. Pharmacological and Genetic Inactivation of Kinin Receptors in Physiology and Therapeutic

Kinin receptor antagonists have been designed on the basis of the peptide amino-acid sequence with structural modifications resulting in competitive or mixed-type inhibition of the binding of the natural ligands [79,80]. These antagonists are pseudopeptides, resistant to peptidases, administered parenterally. The most widely used B2R antagonist was HOE 140, or icatibant [80], which was eventually developed clinically in angioedema (see below). For B1R antagonism, several compounds with structural analogy to bradykinin and desArg9-bradykinin were designed [79]. The kinin receptor antagonists were used in animal studies for probing the physiological role of kinins. The role of kinins and kinin receptors in diseases was further addressed by studying engineered genetic mouse models of kallikrein, B1R and/or B2R deficiency, when these animal models became available [15,17,18,35,81,82].

### 4.1. Animal Studies

Studies with B2R antagonists and B2R deficient mice helped establishing that in healthy animals, the cardiovascular actions of kinins are B2 mediated. The B2R was also found to play a prominent role in experimental cardiac and renal ischemia, although the B1R may also be involved to some extent, as discussed above.

For the vascular and renal complications of diabetes, evidence for a protective role of kinins and B2R comes from the observation of aggravated diabetic nephropathy in several animal models sharing the absence of B2R activation secondary to either inactivation of the *B2R* gene or reduced bioavailability of endogenous kinins in tissue kallikrein knock-out mice [29] or mice expressing three copies of the *ACE /kininaseII* gene [83]. Only one report claimed that the deletion of the B2R was protective against diabetic nephropathy [84]. No clear explanation has been obtained for this isolated observation, and a mouse-strain specific effect has been evoked [85]. Consistent with a protective action of endogenous kinins and B2R activation in diabetic nephropathy, the beneficial effect of ACE inhibition is suppressed by a B2R antagonist in mice or rats [52,53]. B1R inhibition has not been extensively addressed in the diabetic kidney, but some evidence suggests that B1R may also be involved in nephroprotection [86].

In the diabetic retina, kinin production and B1R activation have been reported as having, inversely, deleterious edematous consequences prevented by B1R inhibition [87,88,89].

A peculiar, unexpected effect of B2R inhibition by icatibant is the improvement of skin wound healing in diabetic mice as discussed above [78]. This may be related in part to the resynchronization of fibroblast and keratinocyte proliferations, which are altered by hyperglycemia during skin layer regeneration. The putative clinical application of this observation is the prevention of foot ulcers in diabetic patients.

### 4.2. Clinical Studies in Angioedema

Based on the proposed prominent role of kinins in angioedema, the B2R antagonist Iicatibant was developed for the treatment of this condition. Hereditary angioedema occurs in subjects with C1 inhibitor protein deficiency or functional abnormality, and in some instances in subjects carrying defective mutations of Factor XII [90]. Attacks of angioedema are triggered by unopposed local plasma (pre)kallikrein activation and subsequent kinin release [91]. These attacks may be severe and, if involving the upper respiratory tract, lethal by asphyxia. Other vascular permeability mediators, such as activated complement factors, are also probably involved in angioedema.

Icatibant, administered subcutaneously, was shown in a randomized controlled trial to have beneficial effect in attacks of angioedema, accelerating recovery [92]. The drug has subsequently been approved for the treatment of attacks in patients suffering from hereditary angioedema. Other trials have also supported the benefit of the treatment [93].

However, angioedema can also be acquired and is a well-documented side effect of ACE/kininase II inhibitor treatment, as mentioned above, occurring rarely but more frequently in African American subjects. Angioedema was especially frequent in phase III trials of the mixed ACE/neprilysin inhibitor, omapatrilat. Inhibition of kinin degradation by ACE/kininase II and also, in the case of omapatrilat, by neprylisin is involved in these drug-induced angioedemas.

In ACE inhibitor-induced angioedema, a clinical trial and several observational studies initially suggested the therapeutic activity of icatibant [94], but this was not confirmed in subsequent randomized trials [95,96,97]. Difficulties in the conduct of the first trial, timing of prescription relative to onset of attack and role of associated symptomatic treatments may perhaps explain the discrepancy among the trials. A role of B1R in ACE inhibitor-induced edema could also be hypothesized. This is, however, speculative and not supported by animal studies or observations made in hereditary angioedema. Finally, ACE substrates other than kinins may also be involved, but this is equally speculative.

## 5. Conclusions and Perspectives

Kinins are produced by tissue kallikrein in physiological condition and involved in arterial and renal function, especially in the control of blood flow delivery to organs. These physiological actions of kallikrein and kinins have been documented in both mice and humans and are B2R mediated.

Deficiency in tissue kallikrein and kinins in humans or mice results in minor defective arterial and renal phenotypes in resting condition [19]. However, in pathological situations, such as ischemia, diabetes or hypertension, this deficiency has major consequences for organ damage. Kinins exert cellular actions resulting in endothelium activation, limitation of oxidative stress and stimulation of angiogenesis that eventually afford end-organ protection. This has been well documented experimentally in the ischemic and/or diabetic heart and kidney. On the other hand, excess kinin formation and B2R activation can occur, caused by inappropriate activation of plasma (pre)kallikrein or pharmacological inactivation of kininases, and result in angioedema or hypotension. Angioedema attacks can be treated by pharmacological B2R blockade.

In ischemia and diabetes, kinin release is stimulated and synthesis of B1R and B2R is induced. The issue of the relative role of B1R and B2R in the effects of kinins on diseases remains partly unresolved. Large evidence points toward a prominent role of B2R, but B1R may also be involved. Interestingly, when B2R signaling is inactivated, as occurs, for example, in the heart in diabetes, B1R synthesis is induced and B1R takes over cardio- or vasculo-protective signalization. Thus, contrary to other peptide systems, such as the renin–angiotensin or vasopressin systems, where different receptors mediate distinct peptide actions, or the adrenomedullin system where cooperation between different receptors is required for biological activity, the kallikrein–kinin system displays potential physiological redundancy at the receptor level. A phenomenon of heterodimerization between B2R and the angiotensin II AT1 or AT2 receptors has been reported, but the physiological importance of this proposed molecular interaction remains unestablished [60,98].

Given the beneficial actions of kinins in cardiovascular and renal diseases, the pharmacological activation of kinin receptors has potential therapeutic application. Specific agonists of either B1R or B2R have been synthesized and tested in experimental diseases, with favorable effects (Table 1). These studies have provided proof of concept for therapeutic action of pharmacological B1R or B2R activation but should be translated with caution to human pathological situations. For B2R agonism, unwanted effects, such as angioedema, might occur, although this could be a matter of potency and dosage. For B1R agonism, which consistently displayed therapeutic efficacy in the diabetic ischemic heart, brain or hindlimb, risk of angioedema may be low or absent. The role, if any, of B1R in angioedema has not been addressed. It is interesting to note that angioedema attacks are improved by treatment with icatibant, a specific B2R antagonist, which may, inversely, favor kinin-triggered B1R activation. The administration of a B1R agonist (or as a matter of fact a B2R agonist) for two weeks in mice at therapeutic dosages did not induce detectable unwanted effects, including hypotension or edemas [77,78]. These considerations taken together support the development of B1R agonists for treating diabetic cardiovascular and renal diseases.

## Figures and Tables

**Table 1:** Summary of therapeutic effects of pharmacological B1 or B2 receptor agonists and antagonists in cardiovascular diseases.

	B1R Effects	B2R Effects
	**Agonists**	
**Experimental**	B1R agonist reduces heart infarct size in diabetic mice [71]	B2R agonist acutely but not chronically reduces blood pressure [71]
	B1R agonist enhances peripheral post-ischemic angiogenesis in diabetic mice [77]	B2R agonist reduces heart infarct size in non-diabetic mice [71,74]
	B1R agonist increases blood–brain barrier permeability in mice [73]	B2R agonist enhances peripheral post-ischemic angiogenesis in diabetic mice [77]
	B1R agonist reduces brain infarct size in diabetic mice [76]	B2R agonist opens blood brain barrier in mice [72]
	**Antagonists**	
**Experimental**	B1R antagonist inhibits retinal inflammation in diabetic rats [89]	B2R antagonist improves skin wound healing in diabetic mice [78]
**Clinical**		B2R antagonist accelerates clinical recovery in attacks of hereditary angioedema [92,93]
